# Moderate exercise training does not prevent the reduction in myocardial L‐type Ca^2+^ channels protein expression at obese rats

**DOI:** 10.14814/phy2.13466

**Published:** 2017-10-16

**Authors:** Vitor L. da Silva, Ana P. Lima‐Leopoldo, Artur J. T. Ferron, Jóctan P. Cordeiro, Paula P. Freire, Dijon H. S. de Campos, Carlos R. Padovani, Mário M. Sugizaki, Antonio C. Cicogna, André S. Leopoldo

**Affiliations:** ^1^ Department of Internal Medicine São Paulo State University Botucatu Brazil; ^2^ Center of Physical Education and Sports Department of Sports Federal University of Espírito Santo Vitória Brazil; ^3^ Department of Morphology São Paulo State University Botucatu Brazil; ^4^ Department of Biostatistics Institute of Biosciences São Paulo State University Botucatu Brazil; ^5^ Institute of Health Science Federal University of Mato Grosso Sinop Brazil

**Keywords:** Cardiac physiology, exercise training, myocardial L‐type Ca^2+^ channels, obesity

## Abstract

Authors have showed that obesity implicates cardiac dysfunction associated with myocardial L‐type calcium channels (LTCCs) activity impairments, as well as moderate exercise training (MET) seems to be an important therapeutic tool. We tested the hypothesis that MET promotes improvements on LTCCS activity and protein expression at obesity induced by unsaturated high‐fat diets, which could represent a protective effects against development of cardiovascular damage. Male *Wistar* rats were randomized in control (C, *n* = 40), which received a standard diet and obese (Ob; *n* = 40), which received high‐fat diet. After 20 weeks, the animals were assigned at four groups: control (C; *n* = 12); control submitted to exercise training (ET; *n* = 14); obese (Ob; *n* = 10); and obese submitted to exercise training (ObET; *n* = 11). ET (5 days/week during 12 weeks) began in the 21th week and consisted of treadmill running that was progressively increased to reach 60 min. Final body weight (FBW), body fat (BF), adiposity index (AI), comorbidities, and hormones were evaluated. Cardiac remodeling was assessed by morphological and isolated papillary muscles function. LTCCs activity was determined using specific blocker, while protein expression of LTCCs was evaluated by Western blot. Unsaturated high‐fat diet promoted obesity during all experimental protocol. MET controlled obesity process by decreasing of FBW, BF, and AI. Obesity implicated to LTCCs protein expression reduction and MET was not effective to prevent this condition. ET was efficient to promote several improvements to body composition and metabolic parameters; however, it was not able to prevent or reverse the downregulation of LTCCs protein expression at obese rats.

## Introduction

Obesity is a chronic metabolic disease characterized by excess fat accumulation in otherwise healthy individuals (Korner and Aronne 2003) and has become the most common metabolic and nutritional disorder in industrial and underdeveloped countries (Diffee 2004). The epidemic of obesity worldwide has increased progressively in the last decades (Leopoldo et al. 2011) being currently considered an important public health problem (O'Brien and Dixon 2002). The reduction in physical activity, increase caloric intake or a combination of both has led to a positive energy balance and has been considered the main factors responsible by marked increase in weight in our society. Furthermore, the obese condition is associated with several comorbidities, such as type 2 diabetes mellitus, dyslipidemia, and cardiovascular diseases (Malnick and Knobler 2007; Leite et al. 2012), which are associated with loss of quality of life and mortality (Locatelli et al. 2014).

Obesity per se is able to trigger the onset and/or to induce important changes in the heart and vascular system that could lead to heart failure (Schram and Sweeney 2008; Leite et al. 2012). Experimental studies have showed that obesity induced by different types of high‐fat and high‐energy diets promote myocardial dysfunction and cardiac remodeling (McMullen and Jennings 2007; Leopoldo et al. 2010; Leopoldo et al. 2011; Lima‐Leopoldo et al. 2014). Current researches in our laboratory have demonstrated that obese rats fed high‐fat diet during 15 weeks develop myocardial dysfunction in baseline and after inotropic and maneuvers (Leopoldo et al. 2010; Leopoldo et al. 2011). Several mechanisms have been suggested as responsible for possible cardiac abnormalities in obese models, among them calcium (Ca^2+^) handling (McMullen and Jennings 2007; Lima‐Leopoldo et al. 2008, 2011; Leopoldo et al. 2010; Leopoldo et al. 2011), major regulatory mechanism of myocardial contraction and relaxation (Bers 2002).

The membrane depolarization by action potential leads to the opening of L‐type channels (LTCCs), which the final aim is the contraction of the heart during systole (Bers 2002). These channels are related to the generation of action potentials and signal transduction events at the cell membrane (Bers 2002). Plasma membrane influx of Ca^2+^ by LTCCs leads to Ca^2+^‐induced Ca^2+^ release, which in turn regulates cardiac contractility (Bers 1991). It has also been demonstrated that alterations of amount and function of these channels have been implicated to various cardiovascular diseases, such as: atrial fibrillation, heart failure, and ischemic disorders (Aggarwal and Boyden 1995; Balke and Shorofsky 1998). Different experimental models show the LTCCs plays a fundamental role in cardiac performance, and any defect of this channel may consequently lead to cardiac dysfunction (Rossner 1991; De Tomasi et al. 2009). Recent study realized in our laboratory showed that myocardial dysfunction caused by obesity after 15 weeks is related to L‐type Ca^2+^ channel activity impairment without significant changes in SERCA2a expression and function as well as L‐type Ca^2+^ protein levels (Leopoldo et al. 2011).

Exercise training (ET) is an important nonpharmacological tool to prevention and treatment of obesity and, consequently, may avoid the development of commorbidities associated with adipose accumulation (Rinaldi et al. 2014). The choice of ET intensity is important to achieve the pre‐established goals. In the obesity, it is not advisable to expose individuals to high intensities of exercise, understanding that in this situation the obese individuals, in consequence of high body weight, could be susceptible to injuries. Thus, studies show that moderate exercise training (MET) is appropriated to this specific group (Paulino et al. 2010; Caponi et al. 2012; Rinaldi et al. 2014). Furthermore, different intensities of ET are useful tool to promote cardioprotection (Dantas et al. 2010; Paulino et al. 2010; Caponi et al. 2012; Rinaldi et al. 2014).

The improvements in response to ET on cardiac function and contractility of cardiomyocytes occurs, among other factors, by enhances of Ca^2+^ handling and increase in sensibility to this ion on myocardium (Diffee et al. 2001; Diffee 2004; Kemi et al. 2005, 2007; Wang et al. 2008; Locatelli et al. 2014). These benefits are identified both physiological (Diffee et al. 2001; Kemi et al. 2005, 2007) and pathological conditions (Medeiros et al. 2008; Paulino et al. 2010). In obese rats, after obesity chronic exposure, authors proposed prevention in cardiac dysfunction by MET associated with attenuating the reduction in expression of regulatory proteins of Ca^2+^ handling, but the specific effects of exercise training on LTCCs have not been investigated (Paulino et al. 2010). Although some studies have showed that exercise training promotes positive adaptations and increase gene expression of these channels (Wang et al. 2008; Sugizaki et al. 2011), other researchers have proposed that there were no alterations in Ca^2+^ influx current by endurance training (Mokelke et al. 1997).

However, in experimental models of obesity, LTCCs channels responses by ET and the potential underlying mechanisms on myocardial function still need further clarification. Thus, the aim of this study was to test the hypothesis and to verify if MET improves L‐type Ca^2+^ channels activity and increase its protein expression in obese rats by unsaturated high‐fat diets, both of which are essential for normal cardiac function and for to highlight the preventive role of MET.

## Material and Methods

### Animal care

Thirty‐day‐old male *Wistar* rats (70–100 g) obtained from the Animal Center of Botucatu Medical School (Botucatu, São Paulo, Brazil) were housed in individual cages. The environment was controlled in terms of light (12 h light/dark cycle starting at 6 am), clean‐air room temperature (23 ± 3°C), and relative humidity (60 ± 5%). All experiments and procedures were performed in accordance with the *Guide for the Care and Use of Laboratory Animals* published by the National Research Council (1996) and were approved by the Botucatu Medical School Ethics Committee (UNESP, Botucatu, SP, Brazil) and Ethics Committee for the Use of Animals (UFES, Vitoria, ES, Brazil) under 1036‐2013 and 27/2013, respectively.

### Experimental protocol

**Figure 1:**
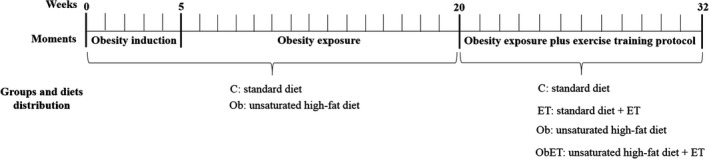
Experimental protocol timeline of 32 weeks. Initially, animals were distributed at two groups, control (C; rats fed with standard diet; *n* = 40) and obese (Ob; rats fed with high‐fat diet; *n* = 40). After 5 weeks of experimental protocol (obesity induction), body weight of C and Ob rats presented statistical difference and this moment was considered the onset of obesity. The obesity exposure period lasted 15 weeks and then the animals were redistributed into more two groups for the practice or not of exercise training maintaining the same diets, composing the following groups: control (C; *n* = 12), control submitted to exercise training protocol (ET; *n* = 14), obese (Ob; *n* = 10), and obese submitted to exercise training (ObET; *n* = 11).

After 7 days of acclimatization, the rats were randomly distributed into two groups: control (C, *n* = 40) and obese (Ob, *n* = 40) (Fig. 1). The C group was fed with a standard diet (*RC Focus 1765*) containing 12.3% of its kcal from fat, 57.9% from carbohydrates, and 29.8% from protein. The Ob animals were fed with four high‐fat diets (RC Focus 2413, 2414, 2415, and 2416), only differing in their flavoring, but not different in micro or macronutrients. The high‐fat diets contained 49.2% of their kcal from fat, 28.9% from carbohydrates, and 21.9% from protein as previously described (Leopoldo and Lima‐Leopoldo 2011). Each diet was changed daily, and the rats were maintained on their respective diets for 32 consecutive weeks. The high‐fat diet was calorically rich (high‐fat diet = 3.65 kcal/g versus low‐fat diet = 2.95 kcal/g) due to its higher fat energy (consisting of saturated and unsaturated fatty acids, which provided 20% and 80% of the fat‐derived calories, respectively). These experimental diets provided sufficient amounts of protein, vitamins, and minerals according to the *Nutrient Requirements of Laboratory Animals* (Benevenga et al. 1995) Animals had free access to water and chow (50 g/day); after 24 h the amount of diet that was not consumed was measured.

### Determination of initial moment and exposure to obesity

The beginning of obesity was determined from the weekly evolution of the body weight of rats. This procedure was utilized to verify the initial moment and, consequently, the duration of obesity. Previous works in our group showed statistical difference in body weight between the groups after the third and fifth weeks of experimental protocol (Lima‐Leopoldo et al. 2014; Ferron et al. 2015). This difference in body weight was confirmed by the increase in fat pads of adipose tissue, among them visceral, epididymal and retroperitoneal fat pads. This moment was regarded as the beginning of obesity (week 5 in this study) (Fig. 1). At this time‐point, the C and Ob rats were maintained on their respective diets for an additional 15 consecutive weeks.

### Characterization of obesity, composition, and redistribution of groups

Body weight (BW), total body fat (BF), and adiposity index (AI) were measured to assess obesity. After starting the experimental protocol, body weight was recorded weekly. At the end of experimental protocol (32 weeks), the animals had been anesthetized with intramuscular injection of ketamine (50 mg/kg) and xylazine (0.5 mg/kg), decapitated, and thoracotomized, the adipose tissue fat pads were dissected and weighed. AI was calculated with the following formula: AI = [total body fat (BF)/final BW] × 100. BF was measured from the sum of the individual fat pad weights as follows: BF = epididymal fat + retroperitoneal fat + visceral fat (Leopoldo and Lima‐Leopoldo 2011; Ferron et al. 2015). At the end of the protocol of exposure of obesity, 15 weeks of initial moment at obesity, was performed the composition and redistribution of rats. In order to constitute two homogeneous groups, ensuring that C group only of animals with characteristics of control animals and Ob group was composed only of animals with characteristics of obese animals was created a confidence interval of 95% (CI) based on the BW means of control and obese rats. It was applied a separation point (SP) between groups; a BW medium point between C upper limit and Ob lower limit; based in this point, were excluded from C group animals with BW above the SP and from Ob group animals with BW below the SP. Thus remained in the study 26 animals from C group (C; *n* = 26) and 21 animals from Ob group (Ob; *n* = 21). After the composition of the experimental groups, animals were redistributed in two more groups as the absence or presence of ET. Therefore, in the third stage of experimental protocol (20 weeks), this study was composed by four groups: control (C; *n* = 12), control submitted to exercise training (ET; *n* = 14), obese (Ob; *n* = 10), and obese submitted to exercise training (ObET; *n* = 11) (Fig. 1).

### Exercise testing and moderate exercise training protocol

In the 21th week of experimental protocol was initiated the moderate exercise training protocol (Fig. 1), adapted from Mostarda et al. (2012). Moderate‐intensity exercise training (55–70% of the maximum running speed) was performed on a motor treadmill (Insight Scientific Equipments, Ribeirão Preto, São Paulo, Brazil), 5 days/week during 12 weeks. Exercise duration was progressively increased from 15 to 60 min/day. All training sessions took place during the afternoon (2:00–5:00 pm). All animals were adapted to the procedure (15 min/day; 5 m/min) for 1 week before the beginning of the aerobic training protocol. Subsequently, the trained groups performed a maximal treadmill test with an initial speed of 9 m/min. Every 3 min at speed was increased to 3 m/min until the animal exhaustion (Rodrigues et al. 2007). The exhaust criteria used in assessing to motor treadmill was not the maintenance of the race on speed proposed by 30 sec. The tests were performed in the first, fifth, and seventh weeks of exercise training to determine aerobic capacity and adequate exercise training intensity.

### Comorbidities and hormones associated with obesity

Exercise training presents beneficial effects on comorbidities associated with obesity. Thus, at the end of experimental protocol, systolic blood pressure, glucose tolerance, homeostatic model assessment index, lipid profile, and blood levels of leptin and insulin evaluations were assessed as described previously (Lima‐Leopoldo et al. 2014; Ferron et al. 2015).

### Cardiac remodeling

Cardiac remodeling was measured by morphological analysis post mortem and isolated papillary muscle function from LV.

### Morphological analysis post mortem

The rats were killed and after thoracotomy, the heart, ventricles, and tibia were separated, dissected, weighed, and measured. Cardiac remodeling at the macroscopic level, which identifies the presence or absence of hypertrophy, was determined by analyzing the following parameters: heart weight (HW), left and right ventricles (LV and RV) weights, and their relation with tibia length.

### Myocardial function

Cardiac contractile performance was evaluated by studying isolated papillary muscle from LV as previously described (Cicogna et al. 2000; Leopoldo et al. 2011). The following mechanical parameters were measured from isometric contraction: maximum developed tension (DT [g/mm^2^]), resting tension (RT [g/mm^2^]), positive (+d*T*/d*t* [g/mm^2^/s]) tension derivative and negative (−d*T*/d*t* [g/mm^2^/s]) tension derivative. The mechanical behavior of papillary muscle was assessed under baseline conditions at 2.5 mmol/L Ca^2^ and after the following inotropic and lusitropic maneuvers: increases in extracellular Ca^2+^ concentration (to test their effects on myofilament machinery) and postrest contraction (PRC), mainly related to sarcoplasmic reticulum (SR) storage and release capacity (Riou et al. 1989). Moreover, to determine the activity or performance of L‐type Ca^2+^ channels during the myocyte contractile cycle, L‐type Ca^2+^ channel blocker was employed. The drug was obtained from Sigma‐Aldrich (St. Louis, MO). The evaluation of L‐type Ca^2+^ channel activity was performed using a specific inhibitor, *Diltiazem hydrochloride* (10^−4^ mol/L), in the presence of cumulative Ca^2+^ concentrations (0.5, 1.0, 1.5, 2.0, and 2.5 mmol/L). The result was expressed as mean percent of inhibition (%) (Cicogna et al. 2000). All variables were normalized per cross‐sectional area of papillary muscle (CSA).

### Analysis of myocardial L‐type Ca^2+^ protein content

The myocardial L‐type Ca^2+^ channels quantity in all groups was evaluated from protein expression by Western blot analysis. Briefly, samples were subjected to SDS‐PAGE in 8–12% polyacrylamide gels depending on the molecular weight of the protein. Kaleidoscope prestained molecular weight markers (Bio‐Rad Laboratories, Hercules, CA, USA) were used as references for determining the precise molecular weights. After electrophoresis, proteins were electrotransferred to nitrocellulose membranes (BioRad Biosciences; NJ, USA). Equal loading of samples (50 mg) and transfer efficiency were monitored with 0.5% Ponceau S staining of the membrane. The blotted membrane was blocked (5% nonfat dry milk, 10 mmol/L Tris‐HCl (pH = 7.6), 150 mmol/L NaCl, and 0.1% Tween 20) for 2 h at room temperature and then incubated overnight at 4°C with specific antibody against L‐type Ca^+2^ channel alpha 1C (Anti‐Calcium Channel Antibody; 1:200, Chemicon^®^ International, CA, USA). Binding of the primary antibody was detected with peroxidase‐conjugated secondary antibodies (rabbit or mouse, depending on the protein, for 2 h at room temperature) and developed with an enhanced chemiluminescence reagent detection reagent (Amersham Biosciences, NJ, USA). Quantification was performed using in vivo Imaging system FX PRO (Bruker Corp., Billerica, MA). Targeted bands were normalized to the expression of β‐actin by using an antibody (1:1000) obtained from Santa Cruz Biotechnology (CA, USA).

### Statistical analysis

Data on general characteristics, comorbidities, cardiac remodeling, and protein expression of myocardial L‐type Ca^2+^ channels were reported as mean ± standard deviation (SD). Comparisons between groups were evaluated using two‐way analysis of variance (ANOVA) for independent samples. A repeated‐measures two‐way ANOVA was utilized to evaluate the positive and negative inotropic effects on myocardial function. When significant differences were found (*P* < 0.05), Bonferroni's post hoc test for multiple comparisons were carried out. The level of significance was 5%.

## Results

### General characteristics, comorbidities, and hormones

**Figure 2:**
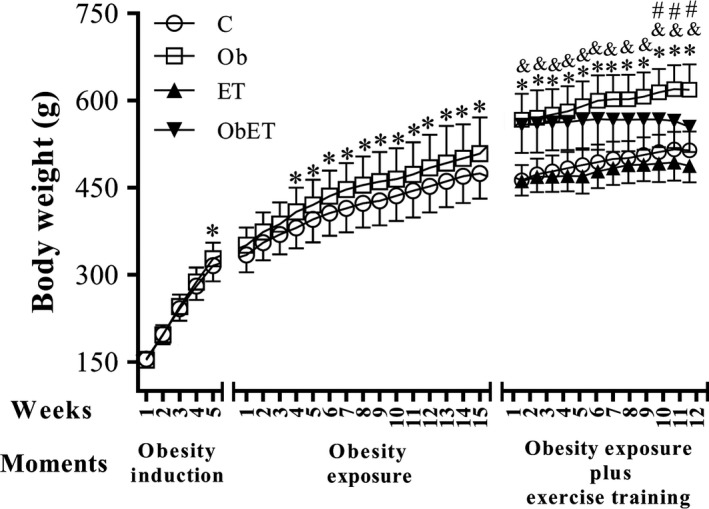
Body weight evolution in obesity induction and exposure, and during the moderate exercise training protocol in control (C; *n* = 12), control submitted to an exercise training (ET; *n* = 14), obese (Ob; *n* = 10), and obese submitted to an exercise training (ObET; *n* = 11) rats. Data are mean ± SD; repeated‐measures two‐way ANOVA and Bonferroni post hoc test. *P < 0.05 – C versus Ob; **P < 0.05 – ET versus ObET; ^†^ *P* < 0.05 – Ob versus ObET.

**Figure 3:**
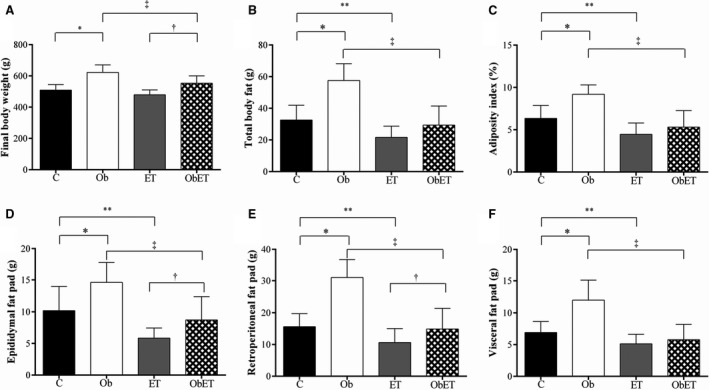
Final body weight (A); total body fat (B), adiposity index (C), epididymal fat (D), retroperitoneal fat (E), and visceral fat (F) pads in control (C; *n* = 12), control submitted to an exercise training (ET; *n* = 14), obese (Ob; *n* = 10), and obese submitted to an exercise training (ObET; *n* = 11) rats after 32 weeks of experimental protocol. Data are mean ± SD; Two‐way ANOVA for independent samples and Bonferroni post hoc test. *P < 0.05 – C versus Ob; **P < 0.05 – C versus ET; ^†^ *P* < 0.05 – ET versus ObET; ^‡^ *P* < 0.05 – Ob versus ObET.

Body weight evolution in obesity induction and exposure, as well as during the exercise training protocol is presented in Figure 2. There was no significant difference observed in all groups for initial body weight. Furthermore, rats became obese at week 5 after initiation of experimental protocol. Figure 2 also shows that there was statistical difference between the groups to body weight since the fourth week until fifteenth week during obesity exposure. Moreover, obese groups, during exercise training protocol, had increased body weight in comparison to their respective controls in all weeks (Fig. 2). Moderate exercise training was able to prevent the body weight gain of ObET animals between tenth and twelfth week of protocol (Fig. 2; *P* < 0.05, Ob > ObET). FBW was elevated (22%) in Ob rats relative to C rats; similar result was observed when compared ObET versus ET (15%; ObET > ET), and Ob versus ObET (12%; Ob > ObET); there was no difference for this variable between C versus ET (Fig. 3A). The consumption of unsaturated high fat‐diet also promoted a substantial elevation of total body fat and adiposity index on Ob in relation to C rats and, and this response was avoided by exercise training (*P* < 0.05, Ob > ObET); besides C rats presented higher values than ET rats (Fig. 3B and C). Specifically, in each fat pad, obesity and ET had opposed effects. ET implicated significant decrease in all fat pads at exercised groups (ET and ObET) when compared with their respective sedentary control groups (C and Ob) (Fig. 3D–F). In addition, ET was able to interrupt the gain of visceral fat, which is considered the most dangerous fat pad for the development of cardiac diseases (ET vs. ObET, *P* > 0.05; Fig. 3F). In all other comparisons among groups, obesity promoted relevant increase in fat pads (C vs. Ob and ET vs. ObET; *P* < 0.05). The comorbidities and hormones associated with obesity and ET are summarized in Table 1. There were no significant differences in the SBP and T‐Chol among all groups. However, ObET rats presented decreased levels to TG when compared with Ob and ET groups. In addition, insulin and leptin levels were significantly affected by obesity and exercise training (Ob > C and Ob > ObET, respectively, *P* < 0.05). Although there was no difference in GL levels under baseline condition (*data not shown*), the area under the curve (AUC) for glucose was significantly greater in ObET rats than ET. There were no significant differences in the AUC between C and Ob rats (Table 1). Nevertheless, HOMA‐IR was significantly affected by exposure to obesity; in addition, ET presented beneficial effects to this variable in obese animals (ObET < Ob, *P* < 0.05).

**Table 1:** Cormobidities, hormones, and cardiac morphology

Variables	C	Ob	ET	ObET
SBP, mmHg	109 ± 12	110 ± 13	112 ± 14	120 ± 12
AUC, mg/dL/min	19 098 ± 2089	20 497 ± 2819	19 671 ± 3142	22 591 ± 3410^b^
HOMA‐IR	12 ± 4	19 ± 9^b^	8.6 ± 3.9	9.2 ± 5.8^c^
TG, mg/dL	53 ± 13	51 ± 8	59 ± 16	37 ± 12^a^, ^b^
T‐Chol, mg/dL	78 ± 26	76 ± 13	77 ± 16	66 ± 18
Insulin, ng/mL	0.84 ± 0.34	1.16 ± 0.45^b^	0.59 ± 0.22	0.62 ± 0.37^c^
Leptin, ng/mL	1.09 ± 0.37	1.91 ± 0.47^b^	0.87 ± 0.41	0.93 ± 0.52^c^
HW, g	1.17 ± 0.08	1.33 ± 0.12^b^	1.22 ± 0.10	1.41 ± 0.23^b^
LVW, g	0.85 ± 0.06	0.96 ± 0.09	0.89 ± 0.07	1.03 ± 0.23^b^
RVW, g	0.22 ± 0.05	0.26 ± 0.03^b^	0.22 ± 0.02	0.27 ± 0.03^b^
HW/Tibia length, g/cm	0.27 ± 0.02	0.30 ± 0.02^b^	0.28 ± 0.02	0.32 ± 0.05^b^
LVW/Tibia length, g/cm	0.20 ± 0.01	0.21 ± 0.02	0.21 ± 0.02	0.23 ± 0.05^b^
RVW/Tibia length, g/cm	0.05 ± 0.01	0.06 ± 0.01^b^	0.05 ± 0.01	0.06 ± 0.01^b^

Data presented as means ± SD. control (C; *n* = 12), control submitted to an exercise training (ET; *n* = 14), obese (Ob; *n* = 10), and obese submitted to an exercise training (ObET; *n* = 11) rats after 32 weeks of experimental protocol. SBP, systolic blood pressure; AUC, area under the curve for glucose; HOMA‐IR, Homeostatic model assessment index TG, triglycerides; T‐Chol, total cholesterol; HW, heart weight; LVW, left ventricle weight; RVW, right ventricle weight; Two‐way ANOVA for independent samples and Bonferroni post hoc test.

a *P *<* *0.05 – C versus Ob.

b *P *<* *0.05 – ET versus ObET.

c *P *<* *0.05 – Ob versus ObET.

### Cardiac remodeling

Table 1 shows the influence of obesity and ET on cardiac remodeling process. Obesity promoted cardiac remodeling after 32 weeks of experimental protocol as visualized by increased values of HW, RVW, HW/tibia length, and RVW/tibia length in relation to C animals. Although there was no statistical difference for LVW, Ob rats presented greater values in relation to C rats (LVW; *P* = 0.062). Furthermore, HW, LVW, RVW, and their relations with tibia length, used as indicators of cell size, were significantly increased in ObET rats compared to ET group, indicating cardiac hypertrophy in this group. Based on these data, when compared exercised to nonexercised groups (C vs. ET and Ob vs. ObET), there was no statistical difference in morphological characteristics, indicating that ET did not lead to myocardial hypertrophy.

### Myocardial function evaluation

**Figure 4:**
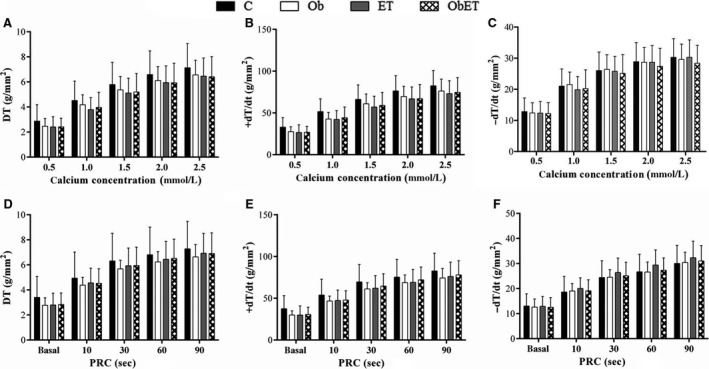
Effects of increasing extracellular Ca^+2^ concentration (A–C) and postrest contraction (D–F) in papillary muscles from control (C), control submitted to exercise training (ET), obese (Ob), and obese submitted to exercise training (ObET). DT, maximum developed tension; +d*T*/d*t*, positive tension derivative and −d*T*/d*t*, negative tension derivative normalized per cross‐sectional area. Data presented as means ± SD. Repeated‐measures two‐way ANOVA (statistical power: 0.893; alpha 0.05).

**Figure 5:**
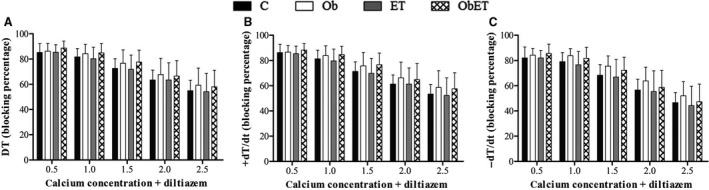
L‐type Ca^+2^ channels activity in papillary muscles from control (C), control submitted to exercise training (ET), obese (Ob), and obese submitted to exercise training (ObET). DT, maximum developed tension; +d*T*/d*t*, positive tension derivative and −d*T*/dt, negative tension derivative normalized per cross‐sectional area. Data presented as mean percent of inhibition (%) ± SD. Repeated‐measures two‐way ANOVA. (statistical power: 0.795; alpha 0.05).

The analysis of myocardial papillary muscle function obtained at baseline condition with Ca^2+^ concentration of 2.5 mmol/L are shown in Table 2. The contraction performance of papillary muscles at baseline was similar for all parameters (DT, RT, +d*T*/d*t*, and −d*T*/d*t*) among the four groups. In addition, the papillary muscle CSA showed no difference between all groups (Table 2). After basal condition, the papillary muscles were submitted to inotropic and lusitropic maneuvers. Figure 4A–C shows that increasing extracellular Ca^2+^ concentration from 0.5 to 2.5 mmol/L, resulting in a positive inotropic effect in myocytes from four groups. However, obesity and ET did not influence on myocardial function. The effects on PRC were consistent with those observed for Ca^+2^ stimulation. Figure 4D–F indicates that PRC induced a significant positive inotropic response in C, ET, Ob and ObET rats after cessation of the stimulus, but the responses were similar among all groups. Figure 5A–C shows the myocardial LTCCs activity from C, Ob, ET, and ObET rats. The maximal inhibition of DT in response to *diltiazem* was 85 ± 7%, 86 ± 6%, 85 ± 6%, and 89 ± 6% at a Ca^2+^ exposure of 0.5 mmol/L in C, Ob, ET, and ObET rats, respectively, but without significant effects between the groups (Fig. 5A). At the same time, *diltiazem* displayed similar negative inotropic behavior on +d*T*/d*t* and −d*T*/d*t* without significant changes among groups (Fig. 5B and C). When analyzed only differences between groups separately, inhibition percentage in response to *diltiazem* was greater in Ob rats than C at a Ca^2+^ exposure of 1.5 and 2.0 mmol/L for all parameters *(data not shown)*. This result suggests that ET was not able to alter the function of LTCCs and increase the myocardial Ca^2+^ entry.

**Table 2:** Baseline isometric contraction

Variables	C	Ob	ET	ObET
DT, g/mm^2^	7.51 ± 0.52	6.91 ± 1.06	7.07 ± 1.48	6.71 ± 1.64
RT, g/mm^2^	0.98 ± 0.11	0.73 ± 0.32	0.84 ± 0.43	0.92 ± 0.30
+d*T*/d*t*, g/mm^2^/s	85.8 ± 22.8	79.3 ± 12.5	77.7 ± 16.2	76.4 ± 17.6
−d*T*/d*t*, g/mm^2^/s	27.7 ± 1.73	26.8 ± 1.90	26.1 ± 1.60	23.6 ± 1.81
CSA, mm^2^	1.02 ± 0.09	1.21 ± 1.00	1.11 ± 0.09	1.25 ± 1.00

Data presented as means ± SD. Control (C), control submitted to exercise training (ET), obese (Ob), and obese submitted to exercise training (ObET); DT, maximum developed tension normalized per cross‐sectional area; RT, resting tension normalized per cross‐sectional area; +d*T*/d*t*, positive tension derivative normalized per cross‐sectional area; −d*T*/d*t*, negative tension derivative normalized per cross‐sectional area; CSA, cross‐sectional area of the papillary muscle. Two‐way ANOVA for independent samples.

### Myocardial L‐type Ca^2+^ (LTCCs) protein expression

**Figure 6:**
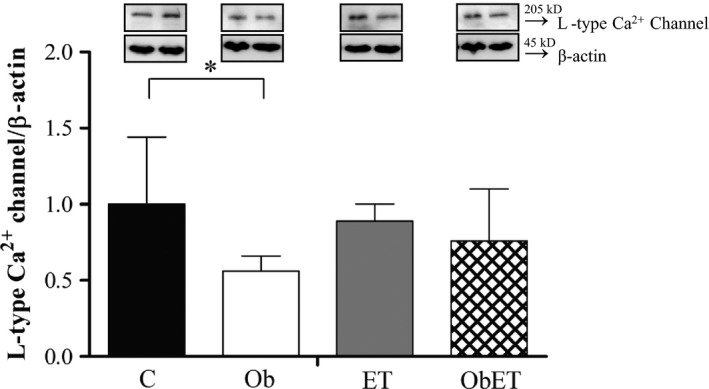
Western blot analysis of L‐type Ca^2+^ channel in cardiomyocytes from control (C), control submitted to an exercise training (ET), obese (Ob), and obese submitted to an exercise training (ObET) rats after 32 weeks of experimental protocol. *n* = 6 per group; Insets: representative gel of L‐type Ca^2+^ channel and β‐actin. Values shown are mean ± standard deviation. Two‐way ANOVA for independent samples and Bonferroni post hoc test. *P < 0.05 – C versus Ob.

The levels of LTCCs were assessed to determine the mechanism for obesity‐induced changes on cardiac function and as ET attenuates or reverses this damage. These results are summarized in Figure 6, which shows that obesity changed the protein levels of LTCCs (C: 1.00 ± 0.44 vs. Ob: 0.56 ± 0.10; *P* < 0.05). ET did not present effects on these abnormalities (Ob vs. ObET; *P* > 0.05). However, it is possible to observe that the obese exercised animals did not had the same response than obese sedentary animals, since, there was not protein levels of LTCCs decrease in the ObET groups in comparison to its respective control (ET vs. ObET; *P* > 0.05), suggesting a positive effect of ET on this alteration promoted by obesity. Additionally, protein levels of L‐type Ca^2+^ channel were similar between the groups C and ET. There was no statistical difference among groups to β‐actin.

## Discussion

This study investigated whether moderate exercise training performed during 12 weeks on rats fed a high‐fat (Ob) could alter the activity and expression of LTCCs and, consequently, attenuates the possible myocardial impairments induced by obesity. The chronic effect of aerobic exercise has been studied in order to further and effectively understand the adaptations caused in humans, as well as an important nonpharmacological intervention to prevent or treat the cardiovascular diseases. This therapeutic approach is potent in obesity, which exerts protective effects against development of cardiovascular damage. However, our results not support the cardioprotective effects of MET on LTCCs activity and protein expression in obese rats, indicating that this nonpharmacological tool failed to attenuate or reverse the myocardial dysfunction promoted by obesity.

Considering that the cardiac overload at obese condition was predominantly volumetric, inducing the eccentric left ventricular to hypertrophy, with increased cardiac chamber without loss of function, the absence of changes induced by MET might be explained by the fact that exercise training causes, generally, few cardiac benefits under healthy conditions in relation to pathological circumstances. Although both physiological and pathological cardiac hypertrophy are associated with an increase in the heart mass, the physiological hypertrophy is associated with a structure and a normal or increased cardiac function (McMullen et al. 2003; McMullen and Jennings 2007; Wisloff et al. 2009; Bernardo et al. 2010; Kehat and Molkentin 2010; Fernandes et al. 2011; Hashimoto et al. 2011; Maillet et al. 2013), whereas the pathological hypertrophy is associated with a complex series of events, including the upregulation of fetal genes, histopathology, and cardiac dysfunction (McMullen et al. 2003; McMullen and Jennings 2007; Wisloff et al. 2009; Bernardo et al. 2010; Kehat and Molkentin 2010; Fernandes et al. 2011; Hashimoto et al. 2011; Maillet et al. 2013). Thus, the MET would be capable of neutralizing the development of pathological cardiac remodeling and acts as therapeutic strategy, treating or reversing to harmful cardiovascular adaptations and, consequently, for controlling to heart failure. Nevertheless, beneficial effects were demonstrated in this study and related to the exercise‐induced improvement of body composition and prevents metabolic disorders produced by HFD. We believe that this is the first study to report the effects of ET on activity and protein expression of L‐type Ca^2+^ channels at myocardium of obese animals.

Experimental models to obesity induction by high‐fat diet have been quite presented in the literature (Relling et al. 2006; Leopoldo et al. 2010; Leopoldo et al. 2011; Lima‐Leopoldo et al. 2011, 2014). In this study, a high‐fat diet rich in unsaturated fatty acid was used. Due to higher caloric contribution than standard diet, high‐fat diet led Ob rats to become obese, increasing all parameters of body composition evaluated. Consistent with previous investigations that used the same diet, total body fat (56%), and adiposity index (69%), variables that analyzed the amount of lipid directly, were substantially greater in Ob rats relative to C rats (Lima‐Leopoldo et al. 2008, 2011, 2014; Leopoldo et al. 2010; Leopoldo et al. 2011). On the other hand, caloric expenditure during ET and the positive chronic influence of an exercise training protocol on body composition, mainly, by decreasing of adipose content are well established. Some authors have shown that aerobic training was effective to decrease adipose tissue area and total adiposity in rats (Friedman, 2009; Haram et al. 2009; Dantas et al. 2010; Machado et al. 2014; Rinaldi et al. 2014). From the period that ET was include on our experimental protocol, the caloric value of high‐fat diet was nullified, whereas, ObET rats showed lipid profile similar to C and ET rats at the end of experiment. These findings suggest that exercise training protocol chosen was able to interrupt obesity progression without dietary changes.

Researchers have shown that obesity and ET exercise training presented inverse effects on glucose metabolism, hormonal levels, blood pressure, and lipid profile (Relling et al. 2006; Haram et al. 2009; Leopoldo et al. 2010; Leopoldo et al. 2011; Caponi et al. 2012; Jodas et al. 2014; Lima‐Leopoldo et al. 2014; Garcia et al. 2016). While ET generates beneficial responses on metabolism as improvements in insulin sensitivity, hypertension reduction, and metabolic control (Haram et al. 2009; Jodas et al. 2014), obesity promotes metabolic disorders such as glucose intolerance, insulin resistance, hypertension, and dyslipidemia (Relling et al. 2006; Leopoldo et al. 2010; Leopoldo et al. 2011; Lima‐Leopoldo et al. 2011) in rodents, resemble the metabolic syndrome ascertained in humans. According Haram et al. (2009), the exercise training is important to correct abnormalities in insulin action and metabolic parameters, being these advantages potentiated by increasing the intensity of activity. Therefore, in obesity, ET has been considered an effective nonpharmacological tool to avoid the development of comorbidities (Gauthier et al. 2004; Machado et al. 2014). Our results demonstrated that ET was efficient to reduce insulin and leptin levels at obese rats, besides, different of sedentary obese (Ob), the exercised obese rats (ObET) did no present insulin resistance. In obese individuals, ET decreases visceral fat and increase fatty acid oxidative capacity and resting fatty acid oxidation; these factors may be related with improve on insulin resistance in obese rats (Caponi et al. 2012; Garcia et al. 2016). Moreover, analyzing cardiac tissue, authors proposed that improvements on insulin resistance by ET in obesity are related with a greater ability of insulin to phosphorylate IR, IRS‐1, IRS‐2, Akt, and Foxo I (Medeiros et al. 2011). Leptin levels, as well documented in literature, decreases in obese individuals through mechanisms activated proportionally to the reduction in fat pads after an exercise training protocol (Gauthier et al. 2004; Paulino et al. 2010; Garcia et al. 2016). Moreover, Garcia et al. (2016) mention authors, which purpose improvements in hypothalamic leptin signaling and increase in leptin receptors on liver and vascular smooth muscle as responsible for leptin levels reduction by chronic exercise training. The reduction in triglicerydes by ET may have been motivated by decreased need for secretion of TG via VLDL (very low‐density lipoprotein) due to diminished delivery of NEFA (nonesterified fat acids) to the liver (Gauthier et al. 2004). Thus, in this study, ET was able to correct alterations on metabolic and lipid profile induced by obesity, including, insulin resistance, and increased blood levels of insulin, leptin, and triglycerides.

According to literature the sedentary lifestyle and reduced aerobic capacity are known predictors of heart failure (Myers et al. 2002), several studies have demonstrated that exercise training exerts a protective effect against ischemic heart disease (Morris et al. 1980; Paffenbarger et al. 1986; Golbidi and Laher 2011; Le Douairon et al. 2014; Hafstad et al. 2015). Researchers have showed the beneficial effects of the physical exercise by improving the functional status of the heart. In this process, several proteins are involved in the mobilization of calcium during the coupling excitation–contraction in the heart among those are sarcoplasmic reticulum Ca^2+‐^ATPase, phospholamban, calsequestrin, sodium–calcium exchanger, ryanodine receptors, and L‐type calcium's channel. Therefore, studies suggest that in severe cases of heart failure may be related due to alterations in the activity and expression of those proteins as well an imbalance in the calcium homeostasis (Beuckelmann et al. 1995; Balke and Shorofsky 1998).

In relation to obesity, the exercise training has therapeutic potential to reduce cardiac *maladaptative* by this condition, since generates cardioprotection in normal (Diffee et al. 2001; Wisløff et al. 2001; Kemi et al. 2005, 2007) and pathological conditions (Medeiros et al. 2008; Powers et al. 2008; Wang et al. 2008; Haram et al. 2009; Paulino et al. 2010), promoting several benefits, such as: physiological hypertrophy (Mokelke et al. 1997; Wang et al. 2008). This physiologic cardiac remodeling occurs by calcineurin deactivation (Yeves et al. 2014) and insulin signaling, and reduce the development of fibrosis in established pathologic hypertrophy (Libonati 2013). Previous findings support the idea that ET improves structure and function cardiac in obese individuals (Cameron et al. 2012). These improvements by ET have been underlying the weight loss, attenuation of hypertension (Haram et al. 2009; Machado et al. 2014), besides decreasing the burden on the heart walls at rest (Locatelli et al. 2014), factors that decrease cardiac overload. Nevertheless, in this study, ET, included in obesity process, without dietary changes, was not able to attenuate the myocardial morphological changes visualized by higher total heart, atrium, right ventricle, and left ventricle in Ob groups. The myocardial hypertrophic process observed in Ob and ObET animals are in agreement with previous findings that have shown cardiac remodeling in rats with high fat diet‐induced obesity (Relling et al. 2006; Leopoldo et al. 2010).

Obesity‐induced cardiac remodeling contributes to cardiac functional abnormalities, resulting in changes in cardiomyocytes and Ca^2+^ handling as visualized by several authors (Relling et al. 2006; Lima‐Leopoldo et al. 2008, 2011, 2014; Leopoldo et al. 2010; Leopoldo et al. 2011). In this study, the obesity did not impair the myocardial function during 32 weeks of experimental protocol, possibly due to protective effect of long‐term obesity by high‐fat diet rich in unsaturated fatty acids (Lima‐Leopoldo et al. 2014). On the other hand, ET improves the cardiomyocyte function in normal conditions (Diffee et al. 2001; Wisløff et al. 2001; Kemi et al. 2005, 2007), prevents deleterious influence of obesity on myocardium by improvements in Ca^+2^ handling, among other factors (Paulino et al. 2010). However, our results demonstrate that there were no alterations in myocardial function at baseline condition and Ca^2+^ handling, after inclusion of ET in obesity process, without dietary changes, even with significant decrease in lipid profile.

Several studies show that obesity is involved with changes in myocardial Ca^2+^ handling protein profile (Lima‐Leopoldo et al. 2008, 2011, 2014). LTCCs have been also implicated as a possible site of adaptation by ET, but in the literature data are conflicting (Mokelke et al. 1997; Wang et al. 2008). There are studies that show ET eliciting adaptations in myocardial L‐type Ca^2+^ channel function and expression (Wang et al. 2008; Sugizaki et al. 2011), but others do not confirm these responses (Mokelke et al. 1997). In this study, the obesity promoted downregulation LTTC without prejudice to activity. Goonasekera et al. 2012 suggested that a reduction in LTCC current leads to neuroendocrine stress, with a SR Ca^2+^ release as a compensatory mechanism in order to preserve the contractility, and therefore resulting in the production of calcineurin/nuclear factor of activated T cells (NFATc) that result in hypertrophy and in the development of cardiac disease; nevertheless, we did not find alterations in myocardial function. In the obesity, researchers have showed myocardial damage associated with LTCCs impairments without decrease in protein expression after 15 weeks of obesity (Leopoldo et al. 2011). In contrast, our results do not demonstrate impairments in LTCCs by obesity, probably due to duration of experimental protocol.

In disagreement with our findings, Leopoldo et al. (2011) showed that, despite the lack of changes in the level of L‐type Ca^2+^ channel protein, the myocardial dysfunction caused by obesity after 15 weeks was related to L‐type Ca^2+^ channel activity impairment. Furthermore, in another study, the obese rats, after chronic exposure to moderate exercise training, presented prevention of reduction in cardiac phosphorylated Thr^17^‐phospholambam and Ser^2808^‐ryanodine (Paulino et al. 2010). In this study, MET presented some punctual positive effect, since in exercise condition there was no reduction in protein expression of L‐type Ca^2+^ channels (ObET vs. ET, *P* > 0.05), which was observed in sedentary obesity condition (Ob vs. C, *P* < 0.05). These results may reinforce the idea that alterations in leptin levels (Ob > ObET, *P* < 0.05) are related to the impairments on protein profile of Ca^2+^ handling as proposed by Paulino et al. (2010). In the blood circulation, high levels of this hormone is involved with cardiac diseases associated with alterations in Ca^2+^ handling protein profile (Paulino et al. 2010).

We believe that time to‐exposure to obesity was not able to affect the myocardial function even with changes in L‐channel expression and, therefore, the MET caring few benefices as increases in calcium sensitivity and cardiomyocyte contractility. It is worth pointing out that our obese animals were killed after 27 weeks of exposure to obesity, because it was necessary that Ob and ObET presented the same experimental time. It is possible that alteration of protein expression have caused an adequacy on cardiac function as an adaptation process by obesity. Thus, in this perspective the cardiomyocytes would present less number of LTCC's, but with normal function; condition that would normalize the myocardial function. However, we know that after long‐term exposition of obesity occurs a maladaptation response, mainly taking into account the large number of changes in body composition, lipid profile and analyzed metabolic variables. Therefore, we believe that all of these alterations, including on body composition and lipid and metabolic profile, could be a signal that these animals in a near future would develop cardiac function alterations. Furthermore, another important confirmation is that the ObET did not present these specific alterations observed in sedentary group, which reduce the possibilities that this group developed any type of myocardial dysfunction.

In summary, obesity promoted metabolic disturbances and myocardial morphological changes. However, the results from this investigation demonstrate that MET did not alter myocardial morphological and functional characteristics, despite modify body composition mainly by the significant decrease in body fat quantity. Obesity caused downregulation from myocardial L‐type Ca^2+^ channel protein expression, in spite of, have not elicited cardiac dysfunction nor impaired on L‐type Ca^2+^ channels activity. In disagreement with our hypothesis, this type of exercise training did not present preventive effects on reduction in myocardial L‐type Ca^2+^ channel protein expression in obese rats. It is interesting that new approaches of exercise training protocols be tested in order to prevent these myocardial alterations verified in obese animals.

There were some limitations of this study that need to be considered. In this study was not evaluated the activity of LTCCs with direct measures as cell electrophysiology, which could verify more rigorously and explain the absence of effects of the moderate exercise training (MET) on downregulation from myocardial L‐type Ca^2+^ channel protein expression, in spite of this study has not elicited cardiac dysfunction nor impaired on L‐type Ca^2+^ channels activity. Second, although we have used the diltiazem on papillary muscle contraction alone with the objective of evaluating Ca^2+^ handling and, consequently, cardiac function, multiple other factors as drug sensitivity, calcium sensitivity and calcium buffering could help in the link between LTCC calcium influx and contraction. Additionally, direct evidence was not provided regarding whether MET improves L‐type Ca^2+^ channels activity and increases its protein expression in obese rats by unsaturated high‐fat diets, which would essential for to highlight the preventive role of MET protocol on protein levels reduction in LTCCs.

## Conflict of Interest

No conflicts of interest, financial or otherwise, are declared by the author(s).
